# Cognitive learning versus practical “hands-on” training for acquisition of laparoscopic surgical skills: an optimal combination study

**DOI:** 10.1007/s00464-025-11673-w

**Published:** 2025-03-27

**Authors:** Amila Cizmic, Nils Schwabe, Frida Häberle, David Killat, Anastasios D. Giannou, Anas A. Preukschas, Anna Nießen, Frank Pianka, Franck Billmann, Arianeb Mehrabi, Beat P. Müller-Stich, Thilo Hackert, Felix Nickel

**Affiliations:** 1 Department of General, Visceral and Thoracic Surgery, University Medical Center Hamburg-Eppendorf, Martinistraße 52, 20246 Hamburg, Germany; 2 Department of General, Visceral, and Transplantation Surgery, Heidelberg University Hospital, Im Neuenheimer Feld 420, 69120 Heidelberg, Germany; 3 Department of Digestive Surgery, University Digestive Healthcare Center Basel, Kleinriehenstrasse 30, 4058 Basel, Switzerland

**Keywords:** Cognitive learning, Practical surgical training, Minimally invasive surgery, Laparoscopy, Cholecystectomy, Novices

## Abstract

**Background:**

Most minimally invasive surgery (MIS) training curricula involve practical training (PT) and cognitive learning (CL) to different extents. It has been proven that acquiring and training specific skills through CL can improve MIS skills. This study aimed to discover the most efficient combination of these two approaches and examine their effects on acquiring MIS skills in novices.

**Methods:**

Sixty medical students without MIS experience participated in this randomized controlled study and were divided into three groups. The first group received the same amount of PT (50%) as CL (50%). The second group focused on PT (75%) compared to the CL (25%). The third group focused more on CL (75%), with less PT implemented (25%). Before and after training, participants performed an ex vivo laparoscopic cholecystectomy (LCHE). Objective Structured Assessment of Technical Skills (OSATS) and Global Operative Assessment of Laparoscopic Skills (GOALS) scores were used for MIS skill evaluation.

**Results:**

Group 1 improved all four performance assessments (global GOALS 14.3 vs. 18.0, *p* < 0.001, LCHE-specific GOALS 5.9 vs. 6.9, *p* = 0.016, global OSATS 19.4 vs. 26.4, *p* < 0.001, LCHE-specific OSATS 37.9 vs. 46.5, *p* = 0.004). Group 2 and Group 3 improved three of four performance scores (Group 2: global GOALS 15.0 vs. 18.4, *p* < 0.001, LCHE-specific GOALS 7.0 vs. 7.7, *p* = 0.115, global OSATS 19.6 vs. 25.8, *p* < 0.001, LCHE-specific OSATS 41.3 vs. 50.7, *p* = 0.001; Group 3: global GOALS 13.8 vs. 17.9, *p* < 0.001, LCHE-specific GOALS 5.8 vs. 6.6, *p* = 0.148, global OSATS 18.9 vs. 25.5, *p* < 0.001, LCHE-specific OSATS 36.8 vs. 43.5, *p* = 0.034).

**Conclusions:**

A balanced combination of PT and CL seems to offer the most effective training compared to predominantly PT or CL training. All three training modes improved MIS skills in novices.

**Supplementary Information:**

The online version contains supplementary material available at 10.1007/s00464-025-11673-w.

Minimally invasive surgery (MIS) has a longer and steeper learning curve than open surgery due to reduced haptic and tactile feedback, three-dimensional vision, and diminished depth perception compared to open surgery [1, 2]. Realizing the necessity for surgeons to acquire proficient MIS skills outside the operation room (OR), simulation-based training tools have been introduced as patient-safe, controllable, and cost-effective alternatives for gaining additional training [3–8]. Many studies have reported that blended learning incorporating interactive learning modules with active tutoring has been proven to deliver an effective and economical approach to learning and teaching skills in the surgical field and other disciplines [2, 9–11].

Most traditional surgical training modalities offer classical practical training (PT) or a hands-on approach [9–12]. These modalities consist of simulation-based training that is focused on technical aspects of the targeted skills, such as dexterity, bimanual in nature, (e.g., grasping, cutting) and procedural skills (e.g., task execution such as suturing and rehearsing scenarios with mannequins). Although undeniably valuable skills, it has been reported that motor-based practice alone does not guarantee surgical expertise [13].

Besides PT, acquiring and training a special set of skills through various cognitive learning (CL) modules has been proven to improve the training of MIS skills [14–16]. One of the most important aspects of the CL is the training of visuospatial and visuomotor cognition, which refers to a group of underlying processes, including spatial visualization, spatial orientation, spatial perception and mental rotation, translation, and scaling, which refers to mental manipulation of two- and three-dimensional images [2]. Furthermore, the mere mental practice of cognitive rehearsal of the MIS surgical task improves performance [17, 18]. CL includes training cognitive strategies like mental readiness and anticipatory planning, which can be trained through preperformance routine, goal setting, and thought management in a surgical simulation training to improve performance [18].

Studies have already reported on the learning effects of various combinations of practical and cognitive surgical training units in medical students. However, these studies only tackled simple surgical tasks such as open and MIS suturing [1, 19]. The effects of different PT and CL combinations on more complex MIS tasks, like performing a complete MIS procedure such as laparoscopic cholecystectomy (LCHE), have not yet been reported. The importance of defining an ideal PT and CL combination in acquiring basic and advanced MIS skills in novices lies in the efficiency and cost-effectiveness of the MIS training.

The primary aim of this study was to investigate the most effective combination of PT and CL and its effects on the learning curve and acquisition of MIS skills in novices using an ex vivo porcine LCHE as a target practice.

## Methods

### Study design

According to the CONSORT guidelines for randomized controlled trials, this study was designed as a prospective, single-center, three-arm, randomized controlled study in a preclinical setting [20]. The study was conducted between September 2022 and June 2023 at the MIS training center at the Department of General, Visceral, and Transplantation Surgery at the University Hospital Heidelberg, Germany. It was part of a facultative MIS training course for medical students during their clinical years. The local ethics committee approved the study (S-436/2018).

### Inclusion and exclusion criteria

The inclusion criteria were medical students (trainees) enrolled at the University Heidelberg Medical School during their clinical years. The exclusion criteria were participation in previous MIS training courses or any experience in MIS.

### The course of the study

**Fig. 1:**
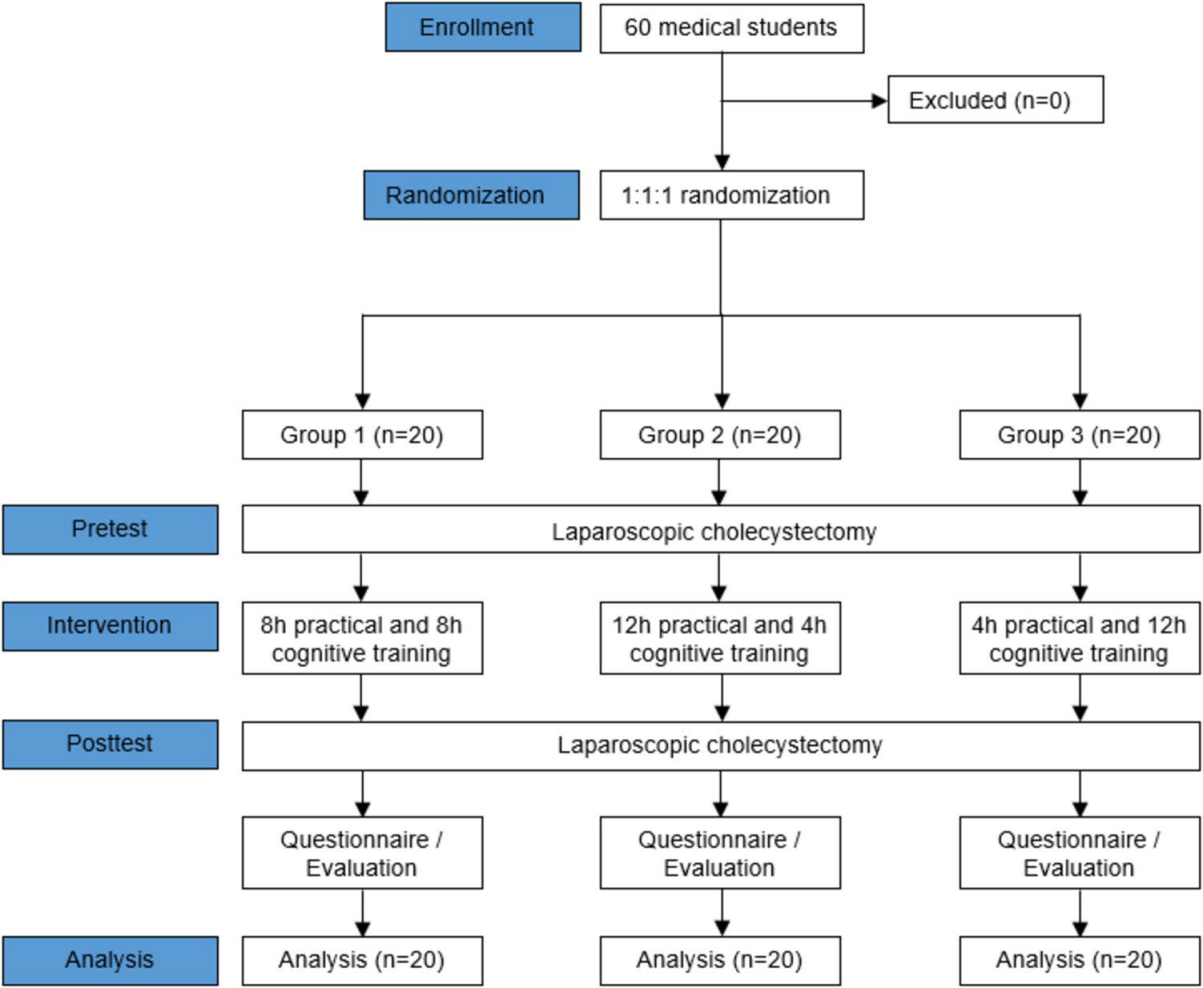
Flow chart of the study design

The trainees were randomized into three groups depending on the percentage of CL and PT they were exposed to (Fig. 1). All groups performed a pre-interventional LCHE on an ex vivo porcine liver. The pre-interventional LCHE was captured on video and evaluated by an independent assessor who did not actively participate in the study.

After the pre-interventional LCHE, the trainees were randomly divided into three groups using a free available randomization tool (https://ctrandomization.cancer.gov/tool/). Group 1 (*n* = 20) had the same amount of CL (50%) and PT (50%), Group 2 (*n* = 20) had more PT (75%) than CL (25%), and Group 3 (*n* = 20) was focused more on the CL (75%) than on PT (25%). Every group had 16 h of predefined CL and PT MIS training (Fig. 1). The percentages of PT and CL in each group represent their respective proportions of the total 16 h of predefined MIS training. Trained tutors guided the students through their entire predefined MIS training, ensuring the MIS training modalities were thoroughly understood and demonstrated. An experienced board-certified surgeon previously trained the tutors in performing and teaching all the MIS training modalities throughout the study course.

After finishing their group-tailored MIS training, each trainee performed a postinterventional ex vivo porcine LCHE. This postinterventional test was video-recorded and later evaluated by the above-mentioned independent assessor.

### Training modalities

The study included a combination of CL and PT MIS training modalities specifically tailored for MIS training in novices.

### Cognitive learning (CL) modules

The CL emphasized training tasks that pose visuospatial problems identical or similar to those faced while learning basic MIS and LCHE skills. It was constructed of six (*n* = 6) training units. All CL modules were undertaken under the supervision of trained tutors to ensure the designated training time was accurate.

1. E-learning programs for LCHE: http://www.webop.de [21] and http://www.websurg.com [22]:

- The trainees were asked to study anatomy, illustrations, and videos of the LCHE procedural techniques.

- Watch "Laparoscopic cholecystectomy: a gold standard case for dissection of Calot's triangle" at http://www.websurg.com [22].

2. Video analysis of performed LCHE with a tutor:

- Trainees analyzed their LCHE with a trained tutor, who pointed out and elaborated on the crucial steps of the procedure, demonstrated the important anatomical structures, explained the usage of the specific instruments, and how to manipulate the surgical field to one's benefit.

3. Situation awareness training through LCHE video assessment with incorporated pop quiz questions was used according to the previous publication (Supplementary Material 1) [23].

4. Annotation of four predefined LCHE video frames:

- Trainees annotated the anatomical structures and instruments in an annotation software (LabelMe) in four predefined LCHE video frames (1: instruments introduced to the surgical field, 2: first cauterization, 3: cystic artery and duct dissected, 4: clipping). This training modality was used according to the previous publication (Supplementary Material 2) [24].

5. A theoretical knowledge test was performed on LCHE procedural questions and intraoperative complications (Supplementary Material 3).

6. Performing LCHE on Touch Surgery mobile serious game application [25].

### Practical training (PT) modules

The PL modules were designed to train fine motoric MIS skills, instrument handling, and camera guidance. Seven (*n* = 7) PL modules were used in the study. Supplementary Material 4 provides a demonstration of the PT modules.

1. Laparoscopic camera guidance:

- The trainees were asked to perform predefined tasks to train their laparoscopic camera guidance skills.

2. Rubber band star:

- The trainees were asked to clamp six rubber bands in a device with six screws.

3. Rubber band attachment:

- The trainees were asked to pull a rubber band into a device made of eyelets and hooks.

4. Cutting out a defined triangle drawn on an index card with laparoscopic scissors.

5. Guiding a surgical needle and thread through loops on a wooden or plastic board in the correct order in a Szabo–Berci–Sackier Box Trainer.

6. Virtual reality (VR) Training:

- Basic module: acquiring basic MIS skills.

- LCHE module: training all individual LCHE steps and the whole LCHE procedure.

7. Tissue dissection on the ex-porcine liver tissue with electrocautery on a Szabo–Berci–Sackier Box Trainer and a standard laparoscopy tower (KARL STORZ GmbH & Co. KG, Tuttlingen, Germany).

### Primary outcome

The study's primary outcome was the MIS skill assessment of the postinterventional LCHE using global and LCHE-specific Objective Structured Assessment of Technical Skills (OSATS) and Global Operative Assessment of Laparoscopic Skills (GOALS) scores (Supplementary Material 5) [26, 27]. Our study utilized both global and task-specific OSATS and GOALS to ensure a comprehensive evaluation of MIS skills by the complementary effect of both performance assessment scores.

### Secondary outcomes

The subjective assessment of the trainees was evaluated using validated scores on current motivation (Questionnaire on Current Motivation (QCM)) and satisfaction with own performance and self-efficacy expectations (General Self-Efficacy Short Scale)). Self-efficacy was assessed using the General Self-Efficacy Short Scale (ASKU), based on a previous publication by Beierlein et al. [28]. The ASKU is an instrument for recording individual competency expectations regarding dealing with difficulties and obstacles in daily life. It is scored using a Likert scale of 1–5, with an average of three items.

The trainees' attention was measured in pre- and postinterventional LCHE with validated psychological tests: the Trail-Making Test (TMT), Frankfurt Attention Inventory 2 (FAIR2), and the Number Link Test (ZVT) [29–31]. The difficulty of each LCHE was assessed by the trained tutors of the MIS section supervising the trainees using the visual analog scale (VAS) [32]. The time required to perform the LCHE was measured in minutes. The complication rate assessment included assessing damage to the liver, cystic duct, cystic artery, gallbladder perforation, and clip placement.

### Statistical analysis

Statistical analysis was performed with SPSS software (version 29, IBM SPSS Inc., Chicago, Illinois, USA). The data were given as absolute frequency and mean ± standard deviation. The t test was used for independent samples in parametric data, and the Mann–Whitney U test was used for independent samples in the case of non-parametric data. For binary endpoints, group differences were calculated using the Chi-square test. A p value of p < 0.05 was considered statistically significant.

## Results

Sixty (*n* = 60) medical students were included in the study. The demographic parameters did not differ between the groups (Table 1). None of the students performed open surgery. Some students assisted and watched open surgical procedures. However, they were equally distributed among three groups (Table 1). As per the inclusion criteria, none of the students had any MIS experience.

**Table 1:** Baseline characteristics of the participants

		Group 1 (*n* = 20)	Group 2 (*n* = 20)	Group 3 (*n* = 20)	p value
Age (years)		23.4 ± 3.7	22.0 ± 2.0	23.0 ± 4,8	0.466
Year of study (n)	3	15 (75.0%)	12 (60.0%)	14 (70.0%)	0.143
	4	4 (20.0%)	8 (40.0%)	3 (15.0%)	
	5	1 (5.0%)	0 (0.0%)	3 (15.0%)	
Dominant hand					
	Right	20 (100.0%)	19 (95.0%)	19 (95.0%)	0.596
Previous open surgery experience					
	Performing	0 (0.0%)	0 (0.0%)	2 (10.0%)	0.126
	Assisting	7 (35.0%)	6 (30.0%)	7 (35.0%)	0.928
	Watching	13 (65.0%)	11 (55.0%)	13 (65.0%)	0.754
Playing video games		6 (30.0%)	5 (25.0%)	7 (35.0%)	0.788
Instrument playing		4 (20.0%)	6 (30.0%)	5 (25.0%)	0.766

*n*, number; Group 1, 50% practical and 50% cognitive training; Group 2, 75% practical and 25% cognitive training; Group 3, 25% practical and 75% cognitive training. Data are presented as numbers (percentage) for categorical variables and mean standard deviation ± for not normally distributed continuous variables. Accordingly, Chi-Quadrat or Fisher's analysis of variance (ANOVA) was used for comparison

### Primary outcome

After their designated training, all three groups improved their global GOALS, global OSATS, and task-specific OSATS scores (Table 2). Only Group 1 improved the task-specific GOALS score (5.9 ± 1.6 vs. 6.9 ± 1.7, *p* = 0.016).

**Table 2:** Performance scores of the pre-/ and postinterventional LCHE

Parameter		LCHE No.1	LCHE No.2	p value
GOALS score global	Group 1	14.3 ± 2.5	18.0 ± 2.8	< 0.001
	Group 2	15.0 ± 1.7	18.4 ± 2.2	< 0.001
	Group 3	13.8 ± 2.8	17.9 ± 2.5	< 0.001
GOALS score task-specific	Group 1	5.9 ± 1.6	6.9 ± 1.7	0.016
	Group 2	7.0 ± 1.6	7.7 ± 1.6	0.115
	Group 3	5.8 ± 1.9	6.6 ± 1.8	0.148
OSATS score global	Group 1	19.4 ± 3.1	26.4 ± 2.6	< 0.001
	Group 2	19.6 ± 2.5	25.8 ± 2.5	< 0.001
	Group 3	18.9 ± 2.8	25.5 ± 3.4	< 0.001
OSATS score task-specific	Group 1	37.9 ± 7.6	46.5 ± 9.5	0.004
	Group 2	41.3 ± 6.9	50.7 ± 8.0	0.001
	Group 3	36.8 ± 10.1	43.5 ± 11.1	0.034

*LCHE*, laparoscopic cholecystectomy; LCHE No.1, pre-test LCHE; LCHE No.2, post-test LCHE; Group 1, 50% practical and 50% cognitive training; Group 2, 75% practical and 25% cognitive training; Group 3, 25% practical and 75% cognitive training; GOALS, global operative assessment of laparoscopic skills; OSATS, objective structured assessment of technical skills. Data are presented as number (percentage) for categorical variables, mean standard deviation ± for normally distributed or median, and [25th and 75th percentile] for not normally distributed continuous variables. Accordingly, the Student’s T-test or Wilcoxon matched-pairs signed rank test was used for comparison

The three groups had no difference in pre-test and post-test performance assessment scores compared to one another. Comparison of the pre- and post-test differences (delta) for each test between the groups revealed no significant differences in delta values (Table 3).

**Table 3:** Comparison of the pre-/ and postinterventional LCHEs between the groups

Parameters		Group 1	Group 2	Group 3		p value
Theoretical Knowledge Test		26.1 ± 2.8	(-)*	27.1 ± 2.1		0.263
LCHE No.1						
GOALS score global	14.3 ± 2.5		15.0 ± 1.7	13.8 ± 2.8	0.266	
GOALS score task-specific	5.9 ± 1.6		7.0 ± 1.6	5.8 ± 1.9	0.066	
OSATS score global	19.4 ± 3.1		19.6 ± 2.5	18.9 ± 2.8	0.720	
OSATS score task-specific	37.9 ± 7.6		41.3 ± 6.9	36.8 ± 10.1	0.212	
LCHE No.2						
GOALS score global	18.0 ± 2.8		18.4 ± 2.2	17.9 ± 2.5	0.804	
GOALS score task-specific	6.9 ± 1.7		7.7 ± 1.6	6.6 ± 1.8	0.149	
OSATS score global	26.4 ± 2.6		25.8 ± 2.5	25.5 ± 3.4	0.572	
OSATS score task-specific	46.5 ± 9.5		50.7 ± 8.0	43.5 ± 11.1	0.067	
Differences in performance scores between the LCHE No.1 and LCHE No.2 (delta)						
GOALS score global	3.7 ± 2.8		3.4 ± 2.4	4.1 ± 3.1	0.725	
GOALS score task-specific	1.0 ± 1.6		0.7 ± 1.9	0.8 ± 2.4	0.889	
OSATS score global	6.9 ± 3.0		6.2 ± 2.8	6.6 ± 4.0	0.802	
OSATS score task-specific	8.6 ± 11.0		9.4 ± 11.0	6.7 ± 13.1	0.758	

*LCHE,* laparoscopic cholecystectomy; Group 1, 50% practical and 50% cognitive training; Group 2, 75% practical and 25% cognitive training; Group 3, 25% practical and 75% cognitive training; (-)*, not performed due to time limitations of designated CL. LCHE No.1, pre-test LCHE; GOALS, global operative assessment of laparoscopic skills; OSATS, objective structured assessment of technical skills; LCHE No.2, post-test LCHE. Data are presented as number (percentage) for categorical variables, mean standard deviation ± for normally distributed or median, and [25th and 75th percentile] for not normally distributed continuous variables. Accordingly, Fisher's analysis of variance (ANOVA) was used for comparison

### Secondary outcomes

**Fig. 2:**
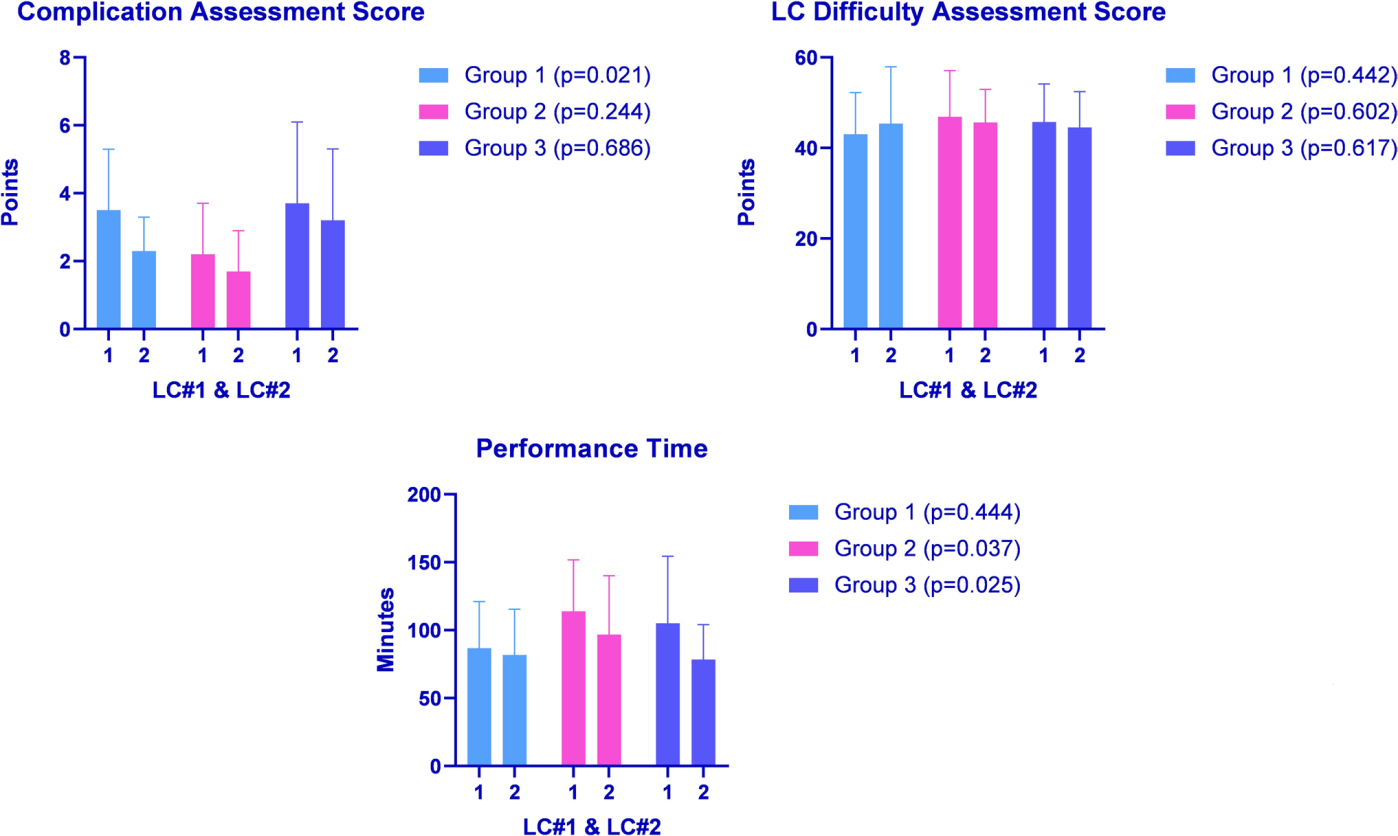
Pre- and postinterventional LCHE comparison regarding complication rate, LCHE difficulty assessment, and performance time

Group 1 significantly reduced the complication rate in the postinterventional LCHE compared to the other two groups. The difficulty assessment of the pre- and postinterventional LCHE was comparable within the groups. Groups 2 and 3 significantly improved postinterventional LCHE performance time (Fig. 2).

There were no differences in pre- and postinterventional ASKU scores. FAIR2, Trail-Marking-Test, and ZVT scores significantly improved in postinterventional LCHE compared to the pre-interventional LCHE in all three groups (Table 4).

**Table 4:** Comparison of the pre-/ and postinterventional psychological test scores

Parameter		LCHE No.1	LCHE No.2	p value
ASKU	Group 1	11.3 ± 1.5	11.8 ± 1.4	0.130
	Group 2	11.9 ± 1.5	12.3 ± 1.7	0.101
	Group 3	11.9 ± 1.1	12.0 ± 1.3	0.098
FAIR2	Group 1	490.3 ± 85.7	568.0 ± 61.2	< 0.001
	Group 2	484.2 ± 64.5	549.8 ± 68.3	< 0.001
	Group 3	472.0 ± 118.5	550.5 ± 118.1	< 0.001
Trail-Marking-Test	Group 1	24.9 ± 6.9	19.2 ± 4.0	< 0.001
	Group 2	25.1 ± 3.9	20.7 ± 4.1	< 0.001
	Group 3	25.6 ± 7.9	20.7 ± 7.5	0.002
ZVT	Group 1	48.4 ± 9.1	44.7 ± 7.7	< 0.001
	Group 2	50.8 ± 6.6	45.8 ± 5.4	< 0.001
	Group 3	51.3 ± 13.3	47.9 ± 15.7	0.002

*LCHE,* laparoscopic cholecystectomy; LCHE No.1, pre-test LCHE; LCHE No.2, post-test LCHE; Group 1, 50% practical and 50% cognitive training; Group 2, 75% practical and 25% cognitive training; Group 3, 25% practical and 75% cognitive training; ASKU; Allgemeine Selbstwirksamkeit Kurzskala (Short Scale for Measuring General Self-Efficacy Beliefs); FAIR2, Frankfurter Aufmerksamkeits-Inventar 2 (Attention Inventory 2); ZVT, Zahlen-Verbindungs-Test (Number Link Test). Data are presented as number (percentage) for categorical variables, mean standard deviation ± for normally distributed or median, and [25th and 75th percentile] for not normally distributed continuous variables. Data are presented as number (percentage) for categorical variables, mean standard deviation ± for normally distributed or median, and [25th and 75th percentile] for not normally distributed continuous variables. Accordingly, the Student's T-test or Wilcoxon matched-pairs signed rank test was used for comparison

**Fig. 3:**
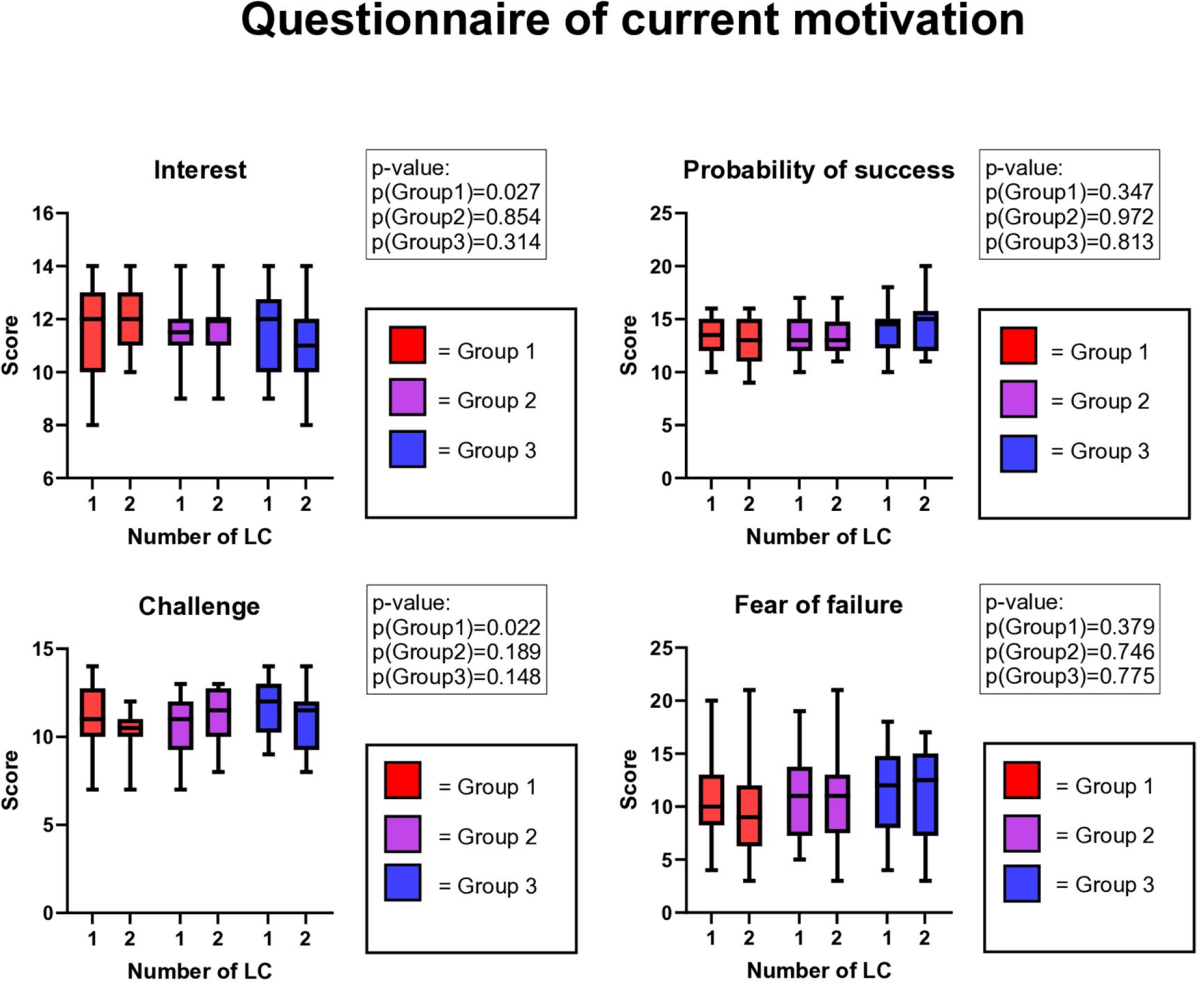
Questionnaire of current motivation (QCM) in pre- and postinterventional LCHE

Group 1 showed increased interest in training and less subjective task challenge in QCM compared to the other two groups (Fig. 3).

## Discussion

The present randomized controlled study showed that MIS training consisting of equal parts of CL and PT improves all LCHE performance scores and reduces complication rates compared to predominantly CL or PT MIS training. Besides improving the technical aspect of the training, the balanced CL and PT MIS training reduced the subjective challenge of performing LCHE. The other two MIS modality combinations (Groups 2 and 3 with CL or PT dominance) improved three out of four postinterventional LCHE performance scores. There were no differences between the three training approaches regarding enhancing performance improvement from the first to the second LCHE (delta). All preoperative assessment scores and demographic characteristics were comparable between the groups. Therefore, the balanced training with equal parts of CL and PT seems to have slight advantages compared to the groups that had either more CL or PT as part of their training.

Optimizing MIS training is crucial due to the known challenges of the MIS learning curve [33–35]. The study aimed to find the ideal MIS training consisting of different parts of CL and PT. However, most studies researching various MIS training modalities focused solely on CL or PT. Combinations of PT and CL have also been used or compared to single-modality training approaches. No study has addressed the ideal combination of the two modalities in MIS training.

The incorporation of structured PT in MIS has been empirically shown to enhance MIS performance, as documented in prior publications [36–41]. Therefore, it was an essential part of the established MIS training program in the presented study. However, training only technical MIS skills can be insufficient to accomplish the full potential of MIS training since PT does not focus on non-technical skills and mental challenges. Hence, the present study combined the two MIS training modules (CL and PT) to demonstrate which combination provides optimal training both objectively and subjectively.

The positive impact of CL on improving technical and non-technical MIS skills has been previously described in trained surgeons and novices [15, 18, 42, 43]. Isreb et al. identified that cognitive non-technical training in LCHE enhances knowledge and hazard awareness in participants. The study was tested for feasibility and included only subjective feedback from the participants on the benefits of CL. Furthermore, the authors developed cognitive training as an online standalone module, demonstrating that CL can benefit even in an unsupervised environment [44]. The present study went even further than supporting the positive subjective feedback reported by Isreb et al. Even the group with predominantly CL (group 3) objectively improved LCHE skills, as shown by OSATS and GOALS skill assessments.

Furthermore, the CL was crucial to improve participants' situational awareness (SA). SA training has proven to reduce errors and enhance cognitive and practical skills in surgery [17, 45]. Kowalewski et al. described the overwhelming benefit of SA training in LCHE performance in surgical novices in a randomized controlled setting [23]. The authors reported that the SA group not only had better LCHE performance but also induced fewer errors than the control group. These findings align with the current study's results, further emphasizing the critical role of CL in enhancing surgical performance and reducing complications.

In the present study, Groups 2 and 3 had reduced their LCHE operation time compared to Group 1. However, Group 1 had the fastest performance time out of the three groups. This indicates that the operation time reduction in Group 1 was not visible due to the faster initial operation time compared to Groups 2 and 3. Furthermore, the discrepancy in operation time reduction could be attributed to the need to improve the complication rate. Only Group 1 had a reduced complication rate in the postintervention test. This could imply that participants in Groups 2 and 3 were faster in the post-test due to a lack of vigilance against the common pitfalls that lead to LCHE complications. Prolonged operation time can lead to postoperative complications [46, 47]. However, Group 2 had the lowest complication rate in pre- and postinterventional LCHE, making the time reduction unnecessary when it comes to reduction of the complication rate.

Nonetheless, even as a valuable parameter of surgical performance, operation time should not be favored over surgical safety and performance quality [48, 49]. This suggests that a balanced MIS training of equal PT and CL parts could aid surgeons in emphasizing surgical safety and precision and minimizing complications. Furthermore, Group 1 had the quickest performance time in the pre-test, which could further influence the lack of time reduction in the post-test compared to the other two groups.

The balanced MIS training containing equal parts of CL and PT (Group 1) resulted in a subjective improvement of interest and decreased sense of procedural challenge compared to predominantly CL or PT training. The effects of the balanced MIS training in this context are invaluable since they can contribute to the subjective workload reduction in MIS [50, 51]. Groups 2 and 3 in the present study did not report these traits after finishing their designated MIS training modules.

## Limitations

Some limitations need to be addressed in the present study. The study occurred in a preclinical setting with medical students as participants. Therefore, the results still need to be transferred to a clinical setting in a real OR since the surgical environment would change, and the surgical residents could have different surgical predispositions. A further limiting factor was using ex vivo porcine liver models for pre- and post-test LCHE. Due to this, there is an imposing question of whether the intervention could benefit the learning curve in human LCHE. The transferability of the results needs to be tested in further feasibility studies in clinical settings with surgical residents or fellows as participants. Furthermore, there is no guarantee that the trainees did not perform additional training, especially in CL modules, such as watching LCHE videos, which could have affected the analyzed results. An additional limitation of the study is that it only engaged one assessor to evaluate LCHE videos. However, the evaluation's objectivity was preserved by ensuring the assessor was blinded and did not actively participate in the study.

## Conclusions

The present study showed that balanced MIS training containing equal CL and PT parts improved LCHE assessment scores and reduced complications. All three groups benefited from their designated MIS training modules containing different parts of CL and PT. Training not only technical but also non-technical skills in MIS shows a promising opportunity to enhance surgical performance and reduce cognitive workload in surgical novices. However, further feasibility studies must validate this assumption in the OR setting and more complex surgical procedures.

## Supplementary Information

Below is the link to the electronic supplementary material.

Supplementary file1 (DOCX 113 KB)

Supplementary file2 (DOCX 756 KB)

Supplementary file3 (DOCX 18 KB)

Supplementary file4 (DOCX 1423 KB)

Supplementary file5 (DOCX 17 KB)
