# Supplementary material for: Cognitive learning versus practical “hands-on” training for acquisition of laparoscopic surgical skills: an optimal combination study
Source: Surg Endosc. 2025 Mar 27;39(5):3068–78. doi: 10.1007/s00464-025-11673-w (PMC12041110; doi:10.1007/s00464-025-11673-w)
Supplement: Supplementary file 1 — Supplementary file1 (DOCX 113 KB) [file 464_2025_11673_MOESM1_ESM.docx]

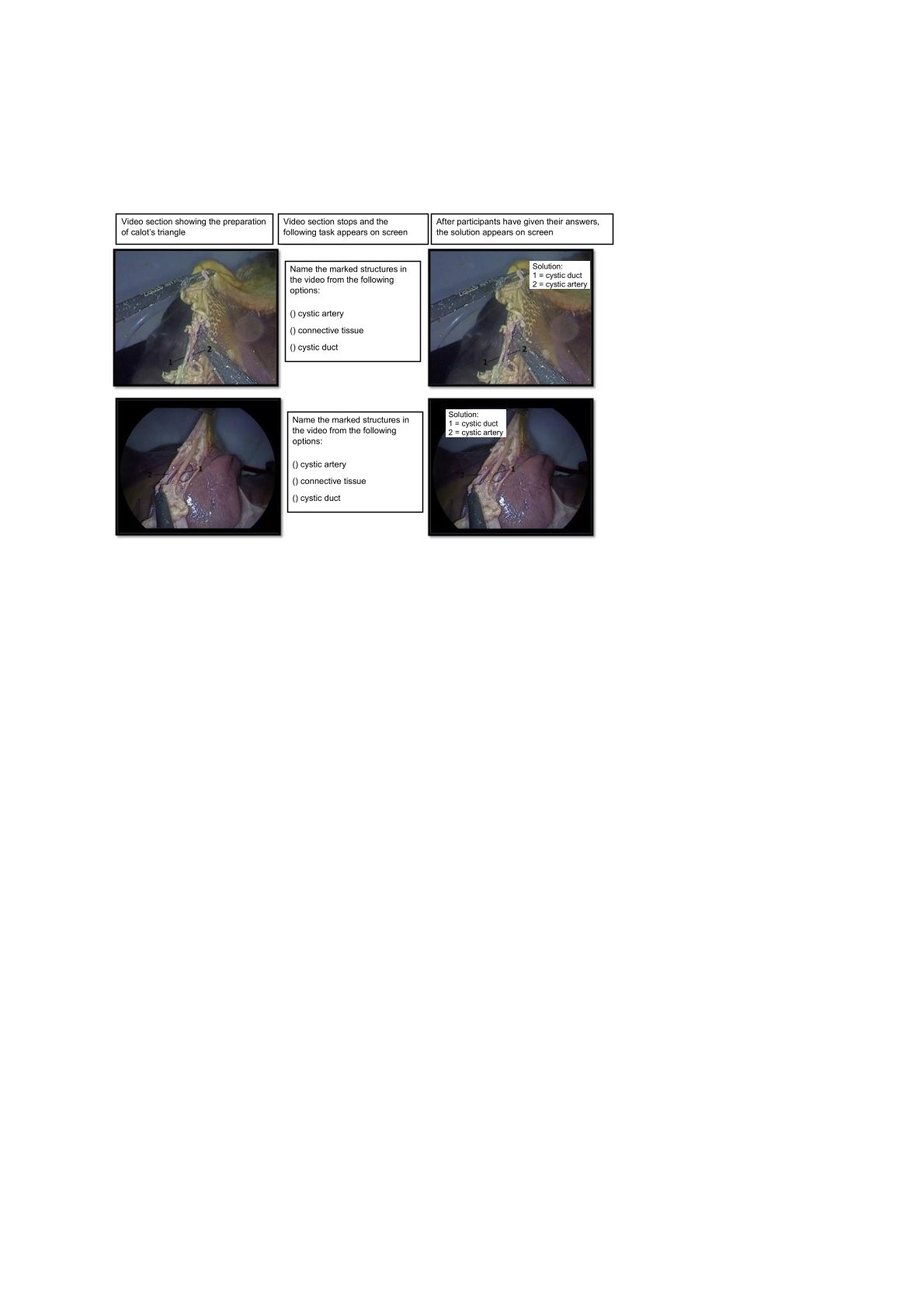


According to Kowalewski et al. [1]

1. Kowalewski, K. F., Seifert, L., Kohlhas, L., Schmidt, M. W., Ali, S., Fan, C., et al. (2023). Video-based training of situation awareness enhances minimally invasive surgical performance: a randomized controlled trial. *Surg Endosc, 37*(6), 4962-4973, doi:10.1007/s00464-023-10006-z.
