# Supplementary material for: Cognitive learning versus practical “hands-on” training for acquisition of laparoscopic surgical skills: an optimal combination study
Source: Surg Endosc. 2025 Mar 27;39(5):3068–78. doi: 10.1007/s00464-025-11673-w (PMC12041110; doi:10.1007/s00464-025-11673-w)
Supplement: Supplementary file 2 — Supplementary file2 (DOCX 756 KB) [file 464_2025_11673_MOESM2_ESM.docx]

According to Cizmic et al. [1]


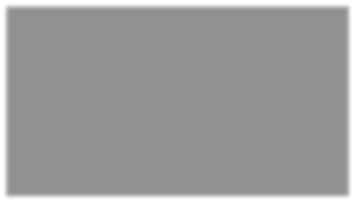

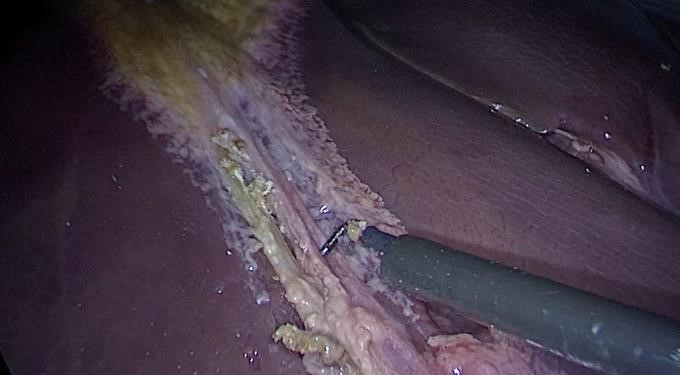

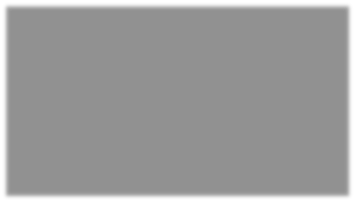

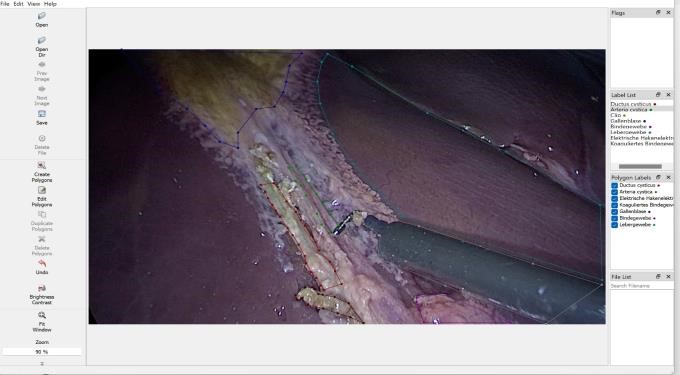

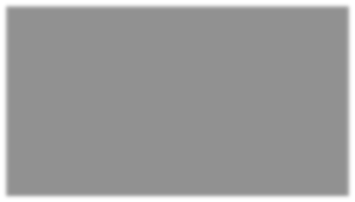

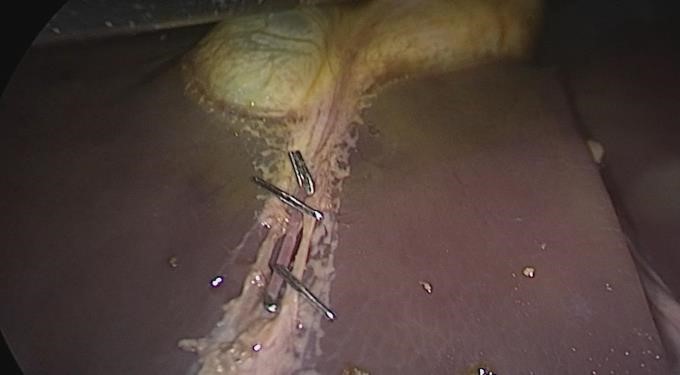

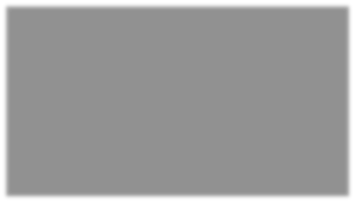

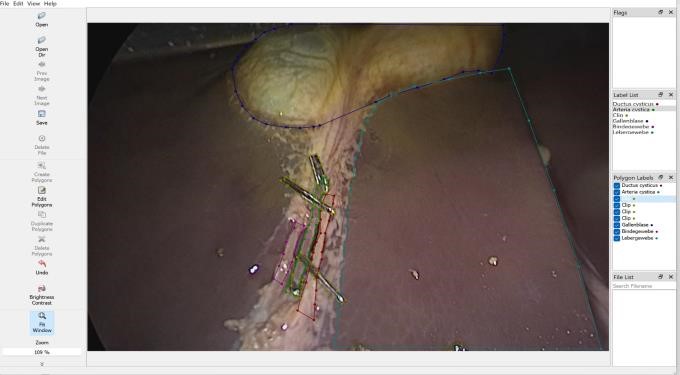

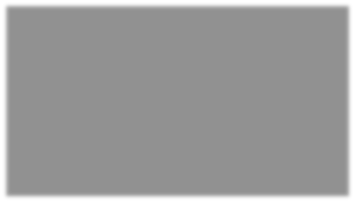

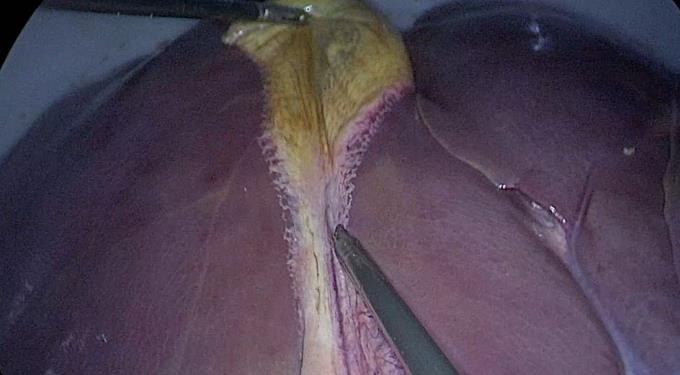

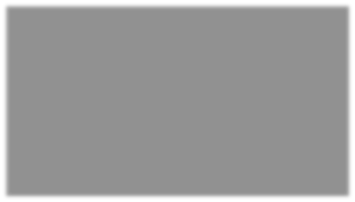

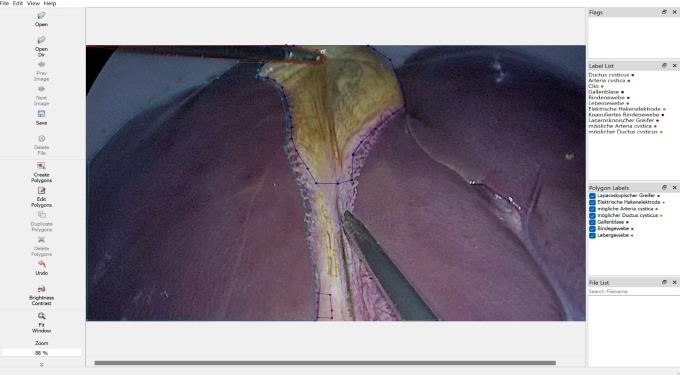


1. Cizmic, A., Häberle, F., Wise, P. A., Müller, F., Gabel, F., Mascagni, P., et al. (2024). Structured feedback and operative video debriefing with critical view of safety annotation in training of laparoscopic cholecystectomy: a randomized controlled study. *Surg Endosc*, doi:10.1007/s00464-024-10843-6.
