# Supplementary material for: Cognitive learning versus practical “hands-on” training for acquisition of laparoscopic surgical skills: an optimal combination study
Source: Surg Endosc. 2025 Mar 27;39(5):3068–78. doi: 10.1007/s00464-025-11673-w (PMC12041110; doi:10.1007/s00464-025-11673-w)
Supplement: Supplementary file 3 — Supplementary file3 (DOCX 18 KB) [file 464_2025_11673_MOESM3_ESM.docx]

**Theoretical Knowledge Test Laparoscopic Cholecystectomy**

1. Which instruments does the surgeon need for a laparoscopic cholecystectomy?

| 1) |
| --- |
| 2) |
| 3) |
| 4) |

1. Which surgical steps are performed in which order during a laparoscopic cholecystectomy?

| 1) |
| --- |
| 2) |
| 3) |
| 4) |
| 5) |
| 6) |
| 7) |

1. What criteria define the Critical View of Safety (CVS)?

| 1) |
| --- |
| 2) |

1. Which 4 main complications can specifically occur during a laparoscopic cholecystectomy?

| 1) |
| --- |
| 2) |
| 3) |
| 4) |

1. Which structures delimit Calot's triangle and at which side do they build the boundary? Which structure runs through Calot's triangle?

| Structure | Which boundary? |
| --- | --- |
| 1) |  |
| 2) |  |
| 3) |  |

Structure that runs through Calot’s triangle:
