# Supplementary material for: Cognitive learning versus practical “hands-on” training for acquisition of laparoscopic surgical skills: an optimal combination study
Source: Surg Endosc. 2025 Mar 27;39(5):3068–78. doi: 10.1007/s00464-025-11673-w (PMC12041110; doi:10.1007/s00464-025-11673-w)
Supplement: Supplementary file 4 — Supplementary file4 (DOCX 1423 KB) [file 464_2025_11673_MOESM4_ESM.docx]

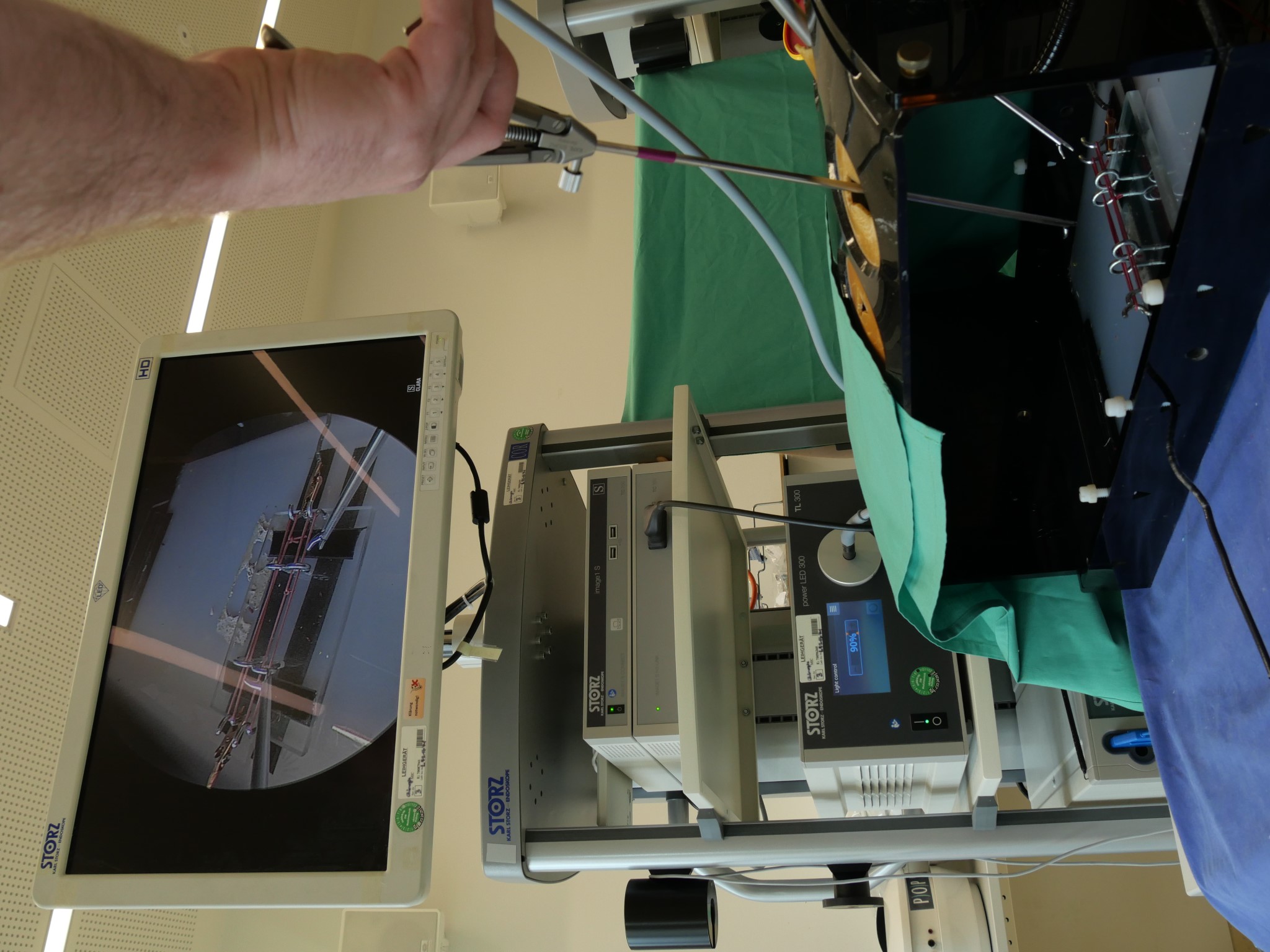

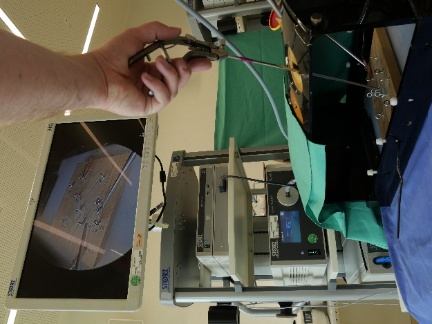

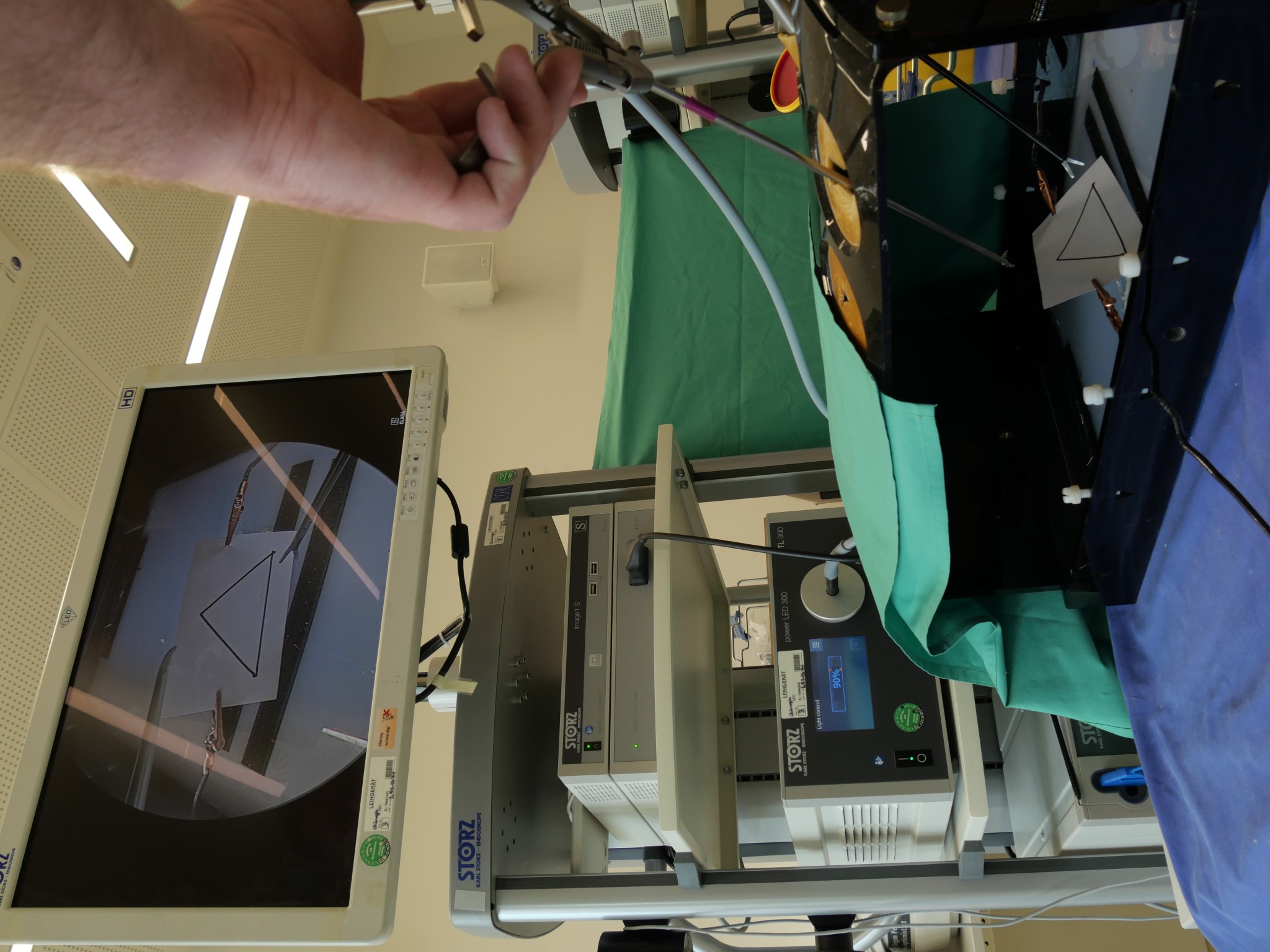

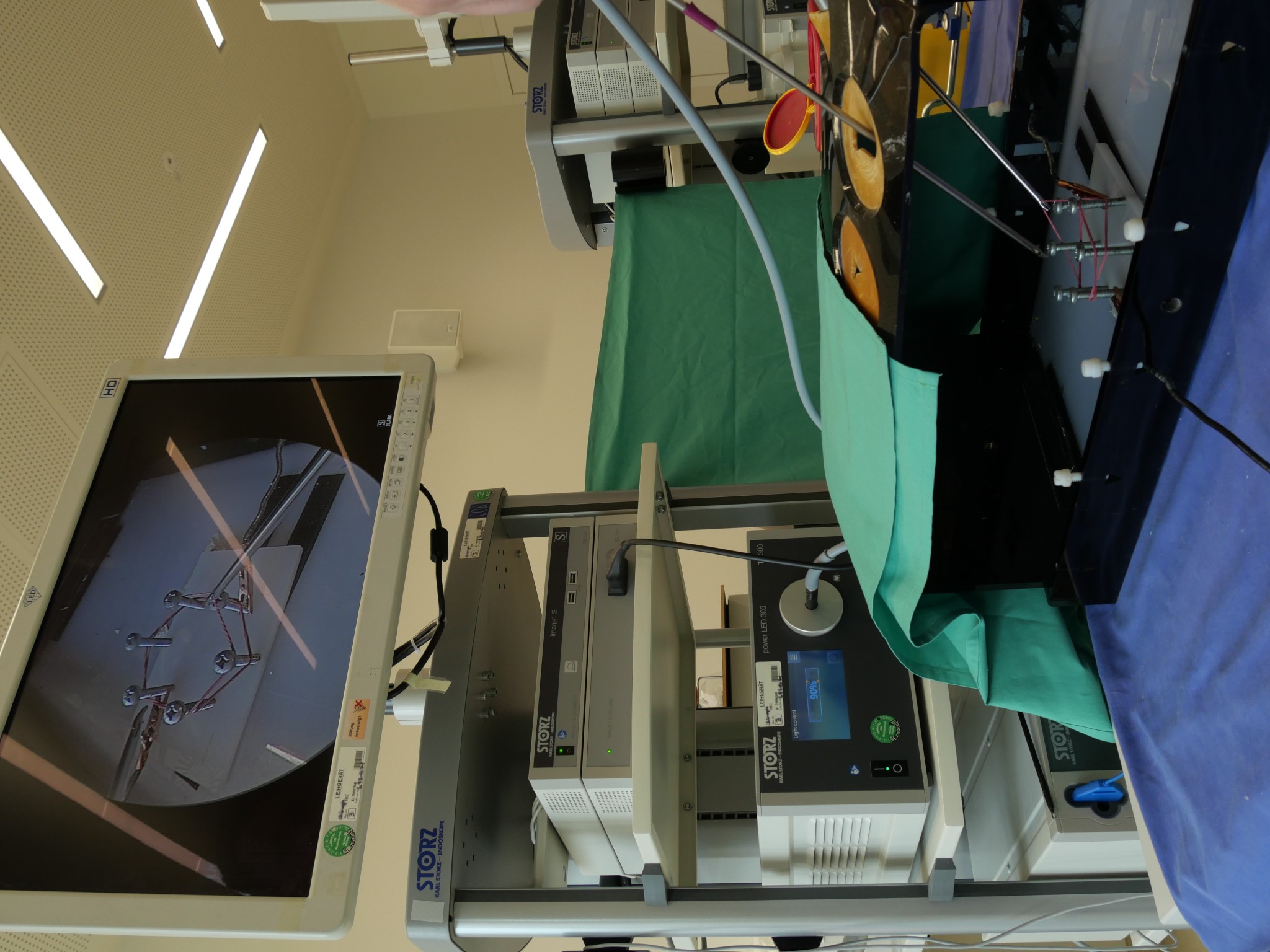
**Supplementary Material 4 – Visual demonstration of PT**

Rubber band star

Cutting a triangle

Needle through loops


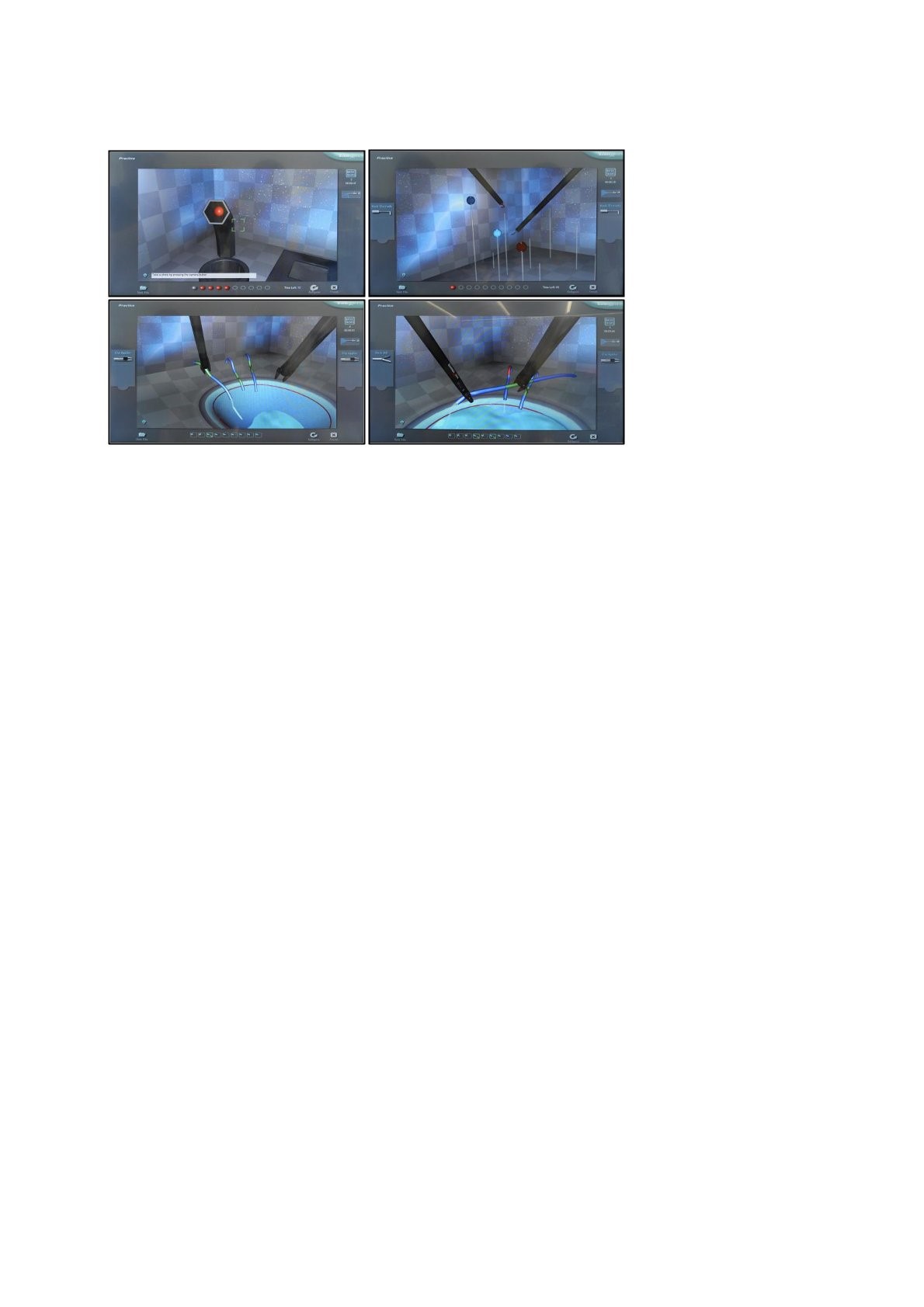


Tasks performed at the Virtual Reality Trainer

Rubber band attachment
