# Supplementary material for: Cognitive learning versus practical “hands-on” training for acquisition of laparoscopic surgical skills: an optimal combination study
Source: Surg Endosc. 2025 Mar 27;39(5):3068–78. doi: 10.1007/s00464-025-11673-w (PMC12041110; doi:10.1007/s00464-025-11673-w)
Supplement: Supplementary file 5 — Supplementary file5 (DOCX 17 KB) [file 464_2025_11673_MOESM5_ESM.docx]

**Supplementary Material 5 – GOALS & OSATS Scores**

**OSATS – specific**

1. Retraction of the gallbladder and 2 – Bad retraction and presentation

presentation of the Calot’s triangle 4

6 – Enough retraction and presentation

8

10 – Experienced retraction and presentation

1. Preparation of the cystic duct 2 - Inadequate

4

6 - Identificated

8

10 – Clearly identified

1. Clipping and separation of 2 – The clips were not correctly placed / Bleeding

the cystic duct 4

6 – The clips were correctly placed (distal and

proximal / No bleeding)

8

10 – The clips were professionally placed (distal and

proximal) with enough distance in between / No

bleeding

1. Preparation of the cystic artery 2 – Inadequate identification and incorrect placement

of the clips

4

6 – Correct identification and correct placement of the

clips

8

10 – Clearly identified, clips correctly placed with

enough space in between

1. Preparation of the gallbladder bed 2 – Perforation of the gallbladder with bile leakage / Stones,

strong bleeding of the liver bed

4

6 – Minimum perforation of the gallbladder, no bile

leakage / Minimal bleeding of the liver bed

8

10 – Intact gallbladder / No bleeding of the liver bed

1. Knowledge about specific operation aspects

2 – Unsecure with important steps of the surgery,

incorrect use of the instruments, not paying

enough attention to the specific anatomy

4

6 – Does the important steps of the surgery, correct

use of the instruments, paying attention to specific

anatomy

8

10 – Secure with all the steps of the surgery,

professional use of the instruments, paying close

attention to specific anatomy

1. Quality of end product 2 – Very bad

4

6 - Satisfying

8

10 – Excellent, over the average

**OSATS global**

1. Tissue respect 1 – Frequently used unnecessary force on

tissue or caused damage by inappropriate

use of instruments

2

3 – Careful handling of tissue but occasionally

caused inadvertent damage

4

5 – Consistently handled tissues appropriately

with minimal damage

1. Time and motion 1 – Many unnecessary moves

2

3 – Efficient time/motion but some

unnecessary moves

4

5 – Economy of movement and maximum

Efficiency

1. Instrument handling 1 – Repeatedly makes tentative or awkward

moves with instruments

2

3 – Competent use of instruments although

occasionally appeared stiff or

awkwardness

4

5 – Fluid movements with instruments and no

awkwardness

1. Knowledge of the instruments 1 – Frequently asked for the wrong instrument

or used an inappropriate instrument

2

3 – Knew the names of most instruments and

used appropriate instrument for the task

4

5 – Obviously familiar with the instruments

required and their names

1. Camera assistance / Use of assistants 1 – Consistently placed assistants poorly or

failed to use assistants

2

3 – Good use of assistants most of the time

4

5 – Strategically used assistant to the best

advantage at all times

1. Flow of operation and forward planning 1 – Frequently stopped operating or needed to

discuss next move

2

3 – Demonstrated ability for forward planning

with steady progression of operative

procedure

4

5 – Obviously planned course of operation

with effortless flow from one move to the next

1. Knowledge of specific procedures 1 – Deficient knowledge. Needed specific

instruction at most operative steps

2

3 – Knew all important aspects of the

operation

4

5 – Demonstrated familiarity with all aspects

of the operation

**GOALS – specific tasks**

0 = not done 1 = done

1. Uses cautery only when all conducting areas are in the O O

field of view

1. Has good control of the instrument O O
2. Grasps gallbladder near clips to begin dissection O O
3. Readjusts tension on gallbladder to optimize exposure O O
4. Avoids dissecting into liver causing undue bleeding O O
5. Avoids perforation of the gallbladder O O
6. Avoids spillage of gallstones O O
7. Maximizes useful dissection in one area before O O

changing approach

1. Performs dissection in appropriate plane the majority O O

of time

1. Obviates the need for surgeon takeover O O

**GOALS global**

1. Depth perception 1 – Constantly overshoots target, wide swings, slow to

correct

2

3 – Some overshooting or missing target, but quick to

correct

4

5 – Accurately directs instrument in the correct plane

to target

1. Bimanual dexterity 1 – Uses only one hand, ignores non dominant hand,

poor coordination between hands

2

3 – Uses both hands, but does not optimize

interaction between hands

4

5 – Expertly uses both hands in a complimentary

manner to provide optimal exposure

1. Efficiency 1 – Uncertain, inefficient efforts; may tentative

movements; constantly changing focus or persisting without origress

2

3 – Slow, but planned movements are reasonably

organized

4

5 – Confident, efficient and safe conduct, maintains

focus on task until it is better performed by way of

an alternative approach

1. Tissue handling 1 – Rough movements, tears tissue, injures adjacent

structures, poor grasper control, grasper frequently slips

2

3 – Handles tissues reasonably well, minor trauma to

adjacent tissue (ie, occasional unnecessary

bleeding or slipping of the grasper)

4

5 – Handles tissues well, applies appropriate traction,

negligible injury to adjacent structures

1. Autonomy 1 – Unable to complete entire task, even with verbal

guidance

2

3 – Able to complete task safely with moderate

guidance

4

5 – Able to complete task independently without

prompting
